# Excess mortality related to the COVID-19 pandemic by age, sex, and cause of death in the Czech Republic

**DOI:** 10.1186/s12889-026-29300-1

**Published:** 2026-09-10

**Authors:** Pavel Piler, Jiří Jarkovský, Vojtěch Thon

**Affiliations:** 1 RECETOX, Faculty of Science, Masaryk University, Kotlarska 2, Brno, 611 37 Czech Republic; 2 Institute of Biostatistics and Analyses, Faculty of Medicine, Masaryk University, Kamenice 3, Brno, 625 00 Czech Republic; 3 Institute of Health Information and Statistics of the Czech Republic, Palackeho namesti 4, Prague, 128 01 Czech Republic

**Keywords:** Excess mortality, Covid pandemic, SARS-CoV-2, Mitochondrial dysfunction, Czech Republic

## Abstract

**Background:**

The aim of the study was to estimate the direct and indirect effects of the COVID-19 pandemic on excess all-cause mortality in the Czech Republic by sex, age group and cause of death.

**Methods:**

National mortality data for the period 2015–2022 were used. For each of the COVID-19 pandemic years (2020–2022), national mortality rates were compared with 2015–2019, and mortality excess was estimated for all causes and selected causes of death separately by sex and age group.

**Results:**

In the COVID-19 pandemic years 2020, 2021 and 2022, the overall excess in all-cause mortality was 15%, 27% and 6%, respectively; excess deaths due to causes other than COVID-19 were 6%, 4% and 1%, respectively. The overall mortality excess was larger in men than women, both for all causes (31% vs. 23% in 2021) and for COVID-19 deaths (relative risk 1.40 in 2021). There was no overall excess mortality in children and adolescents but in the age group 25–49 years, there were more non-COVID-19 deaths in the pandemic years. During the pandemic, there was no mortality increase in three main groups of causes of death (cardiovascular diseases, cancers, respiratory diseases) but there were increases by 20–40% in mortality from heart failure, diabetes mellitus and neurodegenerative diseases.

**Conclusions:**

The large increase in all-cause mortality during the pandemic was driven by deaths coded as COVID-19. The lack of excess mortality from the major groups of chronic diseases suggests that the health system coped well with these conditions. The marked rise in deaths from conditions which have been linked with mitochondrial dysfunction merits further investigation.

**Supplementary Information:**

The online version contains supplementary material available at https://doi.org/10.1186/s12889-026-29300-1.

## Introduction

In the Czech Republic, COVID-19 caused by the SARS-CoV-2 virus was the direct cause of estimated 42,000 deaths (10.8% of all deaths) during the COVID-19 pandemic in 2020–2022. However, the assessment of the impact of the COVID-19 pandemic on overall mortality must also include deaths caused other diseases and conditions related to multiple indirect determinants that affect the functioning of society as a whole. These determinants include the health and social care systems, peoples’ behaviours, and economic and psychosocial conditions [1], which are generally associated with mortality from the main categories of cause of death (i.e., cardiovascular diseases, cancer, and respiratory diseases) [2, 3].

In Europe, several studies have reported excess deaths attributed to the COVID-19 pandemic in selected years, but few studies have reported excess deaths for the entire COVID-19 pandemic period. These studies are mainly from Western Europe and do not include detailed analyses of mortality by cause of death categories [4–10]. The increase in mortality from specific diagnoses may indicate less understood direct mechanisms of action of the pandemic virus. In the case of the SARS-CoV-2 virus, exposure to its active component (spike protein) causes a variety of adverse health effects, some of which can be fatal [11, 12]. For example, recent studies suggest that the mode of action of SARS-CoV-2 exposure is endothelial and mitochondrial dysfunction and associated pathologies that may manifest outside the respiratory system [13, 14].

In this study, we report nationwide excess mortality in all three years of the COVID-19 pandemic period in the Czech Republic (Central Europe). The primary aim of this study is to describe excess deaths associated with each year of COVID-19 pandemic by (a) sex, (b) age, and (c) place of death. The secondary aim is to determine the direct excess deaths from COVID-19, as well as pattern of changes in pandemic mortality by major cause of death categories and by specific diagnoses.

## Methods

### Data

We used weekly data from the National Mortality Database of the Czech Republic on deaths registered in the Czech Republic (10.6 million inhabitants) between 1st January 2015 and 31st December 2022; information available for each death case with individual characteristics: date of death, sex, age, cause of death (classified according to the International Classification of Diseases; ICD-10) and place of death (at home, in hospital or elsewhere). To investigate the impact of COVID-19 pandemic on excess deaths in different age groups the data were aggregated by in the following categories: <12 months, 1–9 years, 10–24 years, 25–49 years, 50–64 years, 65–74 years, 75–84 years, >=85 years.

### Causes of deaths

We used the underlying causes of death from the National Mortality Database. In addition to all-cause mortality, we examined deaths from three major causes of death (cardiovascular diseases, cancers, and respiratory diseases) and deaths from a priori selected causes that could be related to endothelial and mitochondrial dysfunction. Mortality data were extracted from the Czech National Mortality Database for all diseases of the circulatory system (I00-I99), ischaemic heart diseases (I20-I25), cerebrovascular diseases (I60-I69), heart failure (I50), malignant neoplasms (C00-C97), diseases of the respiratory system (J00-J99), pneumonia (J12-J18), chronic lower respiratory diseases (J40-J47), diseases of the digestive system (K00-K93), diseases of the liver (K70-K77), Diabetes mellitus (E10-E15), diseases of the genitourinary system (N00-N39), Parkinson’s disease (G20), other neurodegenerative diseases (G30-G32), intentional self-harm (X60-X84). There were no data available to describe whether the people who died from these causes were infected with SARS-CoV-2. Deaths from COVID-19 were identified as ICD-10 U07.1, U07.2, U09.9 or U10.9.

### Statistical analyses

In this study, excess deaths are defined as the number of deaths above the five-year average. For each pandemic year (2020, 2021, 2022), the average of pre-pandemic deaths in the previous 5 years (2015–2019) was used. This provided a comparison with the number of deaths expected in a typical (non-coronavirus pandemic) year. To quantify the impact of the pandemic on mortality, we calculated the relative risk of excess all-cause deaths (with and without COVID-19 deaths) with 95% confidence intervals for the total population and separately for sex and each age stratum. Crude excess death rates were used in accordance with the findings of Pizzato et al., who demonstrated no significant difference between the age-standardised and crude excess death rates [7].

We also calculated the relative risk of death due to COVID-19 for the male and female population and for selected causes of death. The official estimates of the national population size (from the Czech Statistical Office) were used as the denominators for calculations of mortality rates. All analyses were performed using the ‘Comparing Two Person-Time Rates’ module in a publicly available application at www.openepi.com.

## Results

In the Czech Republic, during the COVID-19 pandemic period (1st January 2020 to 31st December 2022), there were 1208 deaths per 100,000 inhabitants reported in 2020, 1330 in 2021 and 1110 in 2022, while the average mortality rate in 2015–2019 was 1047 per 100,000 inhabitants (Table 1). Table 1 also shows the risks of reported excess deaths during the pandemic period compared with the pre-pandemic period (from 2015 to 2019). The relative risk (RR) of all-cause deaths was 1.15 (1.15–1.16) in 2020, 1.27 (1.26–1.28) in 2021 and 1.06 (1.05–1.07) in 2022. After excluding deaths from/with COVID-19, the risk decreased to 6% in 2020, 4% in 2021, and 1% in 2022. The impact of the pandemic on mortality from all causes was more pronounced in men than in women; the male/female all-cause mortality ratio increased from 1.06 in the pre-pandemic period to 1.09 in 2020, 1.14 in 2021 and 1.09 in 2022 (Table 1). The risk of death from COVID-19 was significantly higher in men than in women, with relative risks as high as 1.40 in 2021 (Table 2).

**Table 1:** Reported all-cause mortality rates during the pandemic period in the Czech Republic and relative risk of all-cause mortality (95% confidence interval) compared to mortality in the last five pre-pandemic years 2015–2019

	Average annual rate 2015–2019*	2020 rate*	RR 2020 vs. prepandemic	2021 rate*	RR 2021 vs. prepandemic	2022 rate*	RR 2022 vs. prepandemic
Total reported deaths	1047	1208	1.15 (1.15–1.16)	1330	1.27 (1.26–1.28)	1110	1.06 (1.05–1.07)
Total reportes male deaths	1079	1263	1.17 (1.16–1.18)	1418	1.31 (1.30–1.33)	1157	1.07 (1.06–1.08)
Total reported female deaths	1015	1155	1.14 (1.13–1.15)	1244	1.23 (1.21–1.24)	1066	1.05 (1.04–1.06)
Reported deaths without deaths from COVID-19	1047	1110	1.06 (1.05–1.07)	1088	1.04 (1.03–1.05)	1055	1.01 (1.00-1.02)
Reported male deaths without deaths from COVID-19	1079	1150	1.07 (1.05–1.08)	1135	1.05 (1.04–1.06)	1095	1.02 (1.00-1.03)
Reported female deaths without deaths from COVID-19	1015	1070	1.05 (1.04–1.07)	1042	1.03 (1.02–1.07)	1016	1.00 (0.99–1.01)

*Rate per 100,000 persons

**Table 2:** Reported death rates (per 100,000 persons) from COVID-19 in the Czech Republic during the pandemic period (2020–2022) by sex

	Total	Females	Males	Relative Risk for males (female = 1.0)
Reported deaths from COVID-19 in 2020	98	85	113	1.33 (1.28–1.38)
Reported deaths from COVID-19 in 2021	242	202	283	1.40 (1.37–1.43)
Reported deaths from COVID-19 in 2022	56	50	62	1.23 (1.17–1.29)

**Fig. 1:**
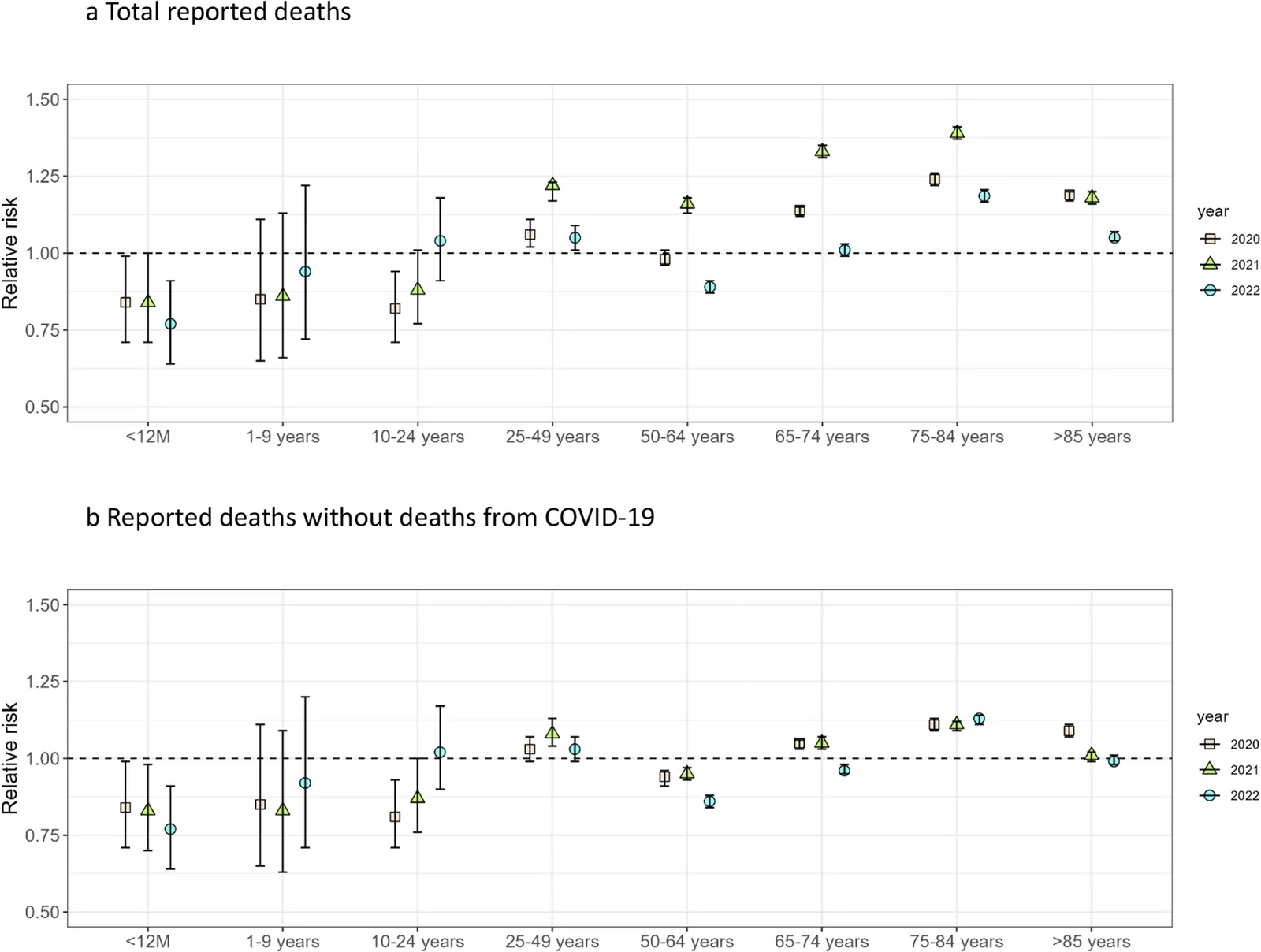
Relative risk deaths in different age groups in 2020 – 2022. **a** Total reported deaths. **b** Reported deaths without deaths from COVID-19

Figure 1 and Supplementary Table 1 show the pandemic risk of excess deaths from all causes in different age groups. In children and adolescents, the pandemic risk of death from all causes (Fig. 1a) and after excluding deaths from/with COVID-19 (Fig. 1b) was lower than in the pre-pandemic period. By contrast, in the 25–49 age group, the excess risk of death during the pandemic was higher than in the pre-pandemic period; even after excluding deaths from/with COVID-19, it was 3% higher in 2020, 8% higher in 2021 and 3% higher in 2022 (Fig. 1b, Supplementary Table 1). As expected, the pandemic risk of death from all causes in the 50 + age group was higher in 2020 and 2021 than in the pre-pandemic period but after excluding deaths due to/with COVID-19, the risk was comparable to pre-pandemic deaths (Fig. 1b).

**Fig. 2:**
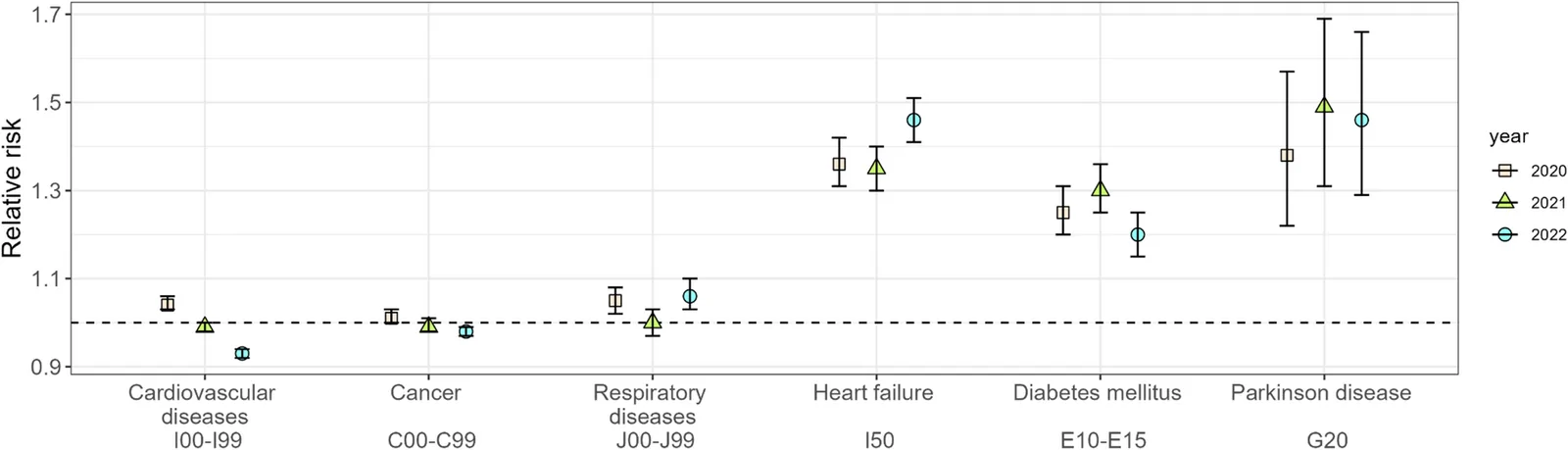
Relative risk of death from specific causes of death

The risks of excess mortality by specific causes of death are shown in Fig. 2, Supplementary Tables 2 and Supplementary Table 3. A 4% excess mortality for all circulatory diseases (ICD-10 I00-99) was observed in the first pandemic year but in the next two pandemic years (2021 and 2022), mortality fell below pre-pandemic levels (Fig. 2). There was no difference between the number of deaths due to malignant tumours during the pandemic and before the pandemic. Unexpectedly, the mortality from respiratory diseases did not show apparent increase in any of the three years of the pandemic. The pandemic risk of death from chronic obstructive pulmonary disease (COPD) decreased compared with the pre-pandemic period. However, the risk of death from pneumonia increased (Supplementary Table 2).

Supplementary Table 2 also shows that mortality from ischaemic heart diseases decreased from 218 deaths per 100,000 inhabitants in 2020 to 208 deaths in 2021 and 183 deaths in 2022. A similar decrease in mortality was observed for cerebrovascular diseases, with an average of 80 deaths per 100,000 inhabitants in 2015–2019, 71 deaths in 2020, 68 deaths in 2021 and 64 deaths in 2022.

Figure 2 shows selected diagnoses for which the risk of mortality increased by more than 20% in all three years of the COVID-19 pandemic compared with the pre-pandemic period. Compared with pre-pandemic period, mortality from heart failure increased by 36% in 2020, 35% in 2021 and 46% in 2022. We also found a significant increase in mortality from metabolic and neurodegenerative diseases. For example, the pandemic excess risk of death from diabetes mellitus was up to 30% higher than in the previous 5 years before the pandemic. Similar changes were found for Parkinson’s disease; the excess risk of death was 38% (RR 1.38; CI 1.22–1.57) in 2020, 49% (RR 1.49; CI 1.31–1.69) in 2021 and 46% (RR 1.46; CI 1.29–1.66) in 2022 (Fig. 2, Supplementary Table 2). There were no major differences in the pattern of mortality excess between men and women (Supplementary Table 3).

Table 3 shows places where people died before and during the pandemic. Compared with the pre-pandemic period, 26% more deaths were reported at home in 2020, 36% more in 2021 and 24% more in 2022. In 2022, however, the number of hospital deaths was similar as before the pandemic.

**Table 3:** Number of deaths by place of deaths before the pandemic (2015–2019) and during the pandemic in the Czech Republic

	Average annual no. of deaths 2015–2019	2020	Change 2020 vs. 2015-19 (%)	2021	Change 2021 vs. 2015-19 (%)	2022	Change 2022 vs. 2015-19 (%)
Hospital deaths	71,242	78,108	↑ (+ 10%)	88,575	↑ (+ 24%)	72,108	↑ (+ 1%)
Home deaths	24,133	30,375	↑ (+ 26%)	32,912	↑ (+ 36%)	29,949	↑ (+ 24%)
Deaths in other places	15,754	20,806	↑ (+ 32%)	18,404	↑ (+ 17%)	18,162	↑ (+ 15%)

## Discussion

This study investigated nationwide excess mortality in each year of the COVID-19 pandemic in the Czech Republic. Apart from the considerable number of deaths reported as being due to COVID-19 (account for 10.8% of all deaths on or with COVID-19 in 2020–2022), we found an excess of deaths from other causes (with an excess of up to 6% in 2020). Several causes of deaths showed marked increases during the pandemic, including heart failure and metabolic and neurodegenerative conditions.

The most recent study by Pizzato and colleagues have provided comprehensive assessment of the impact of COVID-19 on overall mortality in Europe from 2020 to 2023. The study estimated over 1.6 million excess deaths (+ 8%); the excess was notably higher in Eastern European countries (+ 13.2%) compared to Western (+ 6.3%), Northen (+ 7.0%) and Southern Europe (+ 7.8%). This study does not distinguish between deaths from COVID-19 and other causes; it describes the overall excess of deaths in Europe during the pandemic [7]. Other several previous studies have evaluated trends in excess pandemic mortality in the European countries [4–6, 10], but these studies either report deaths from all causes, or only mortality results for one or two pandemic years. We therefore compare our findings primarily with data from the UK Office for Health Improvement and Disparities project [15], which uses similar methods and the same period to our study.

Compared with the previous five pre-pandemic years, there was a substantial excess of deaths in each year of the COVID-19 pandemic in the Czech Republic (15% in 2020, 27% in 2021 and 6% in 2022, compared with 19% in 2020; 9% in 2021 and 6% in 2022 in the United Kingdom. If deaths with/from COVID-19 were excluded, we found a slight excess of deaths (between 1% and 6%) in each pandemic year in the Czech Republic, while in the UK there was no excess mortality deaths in these years after excluding deaths due to COVID-19 [15].

During the three pandemic years, 42,029 COVID-19-related deaths were reported in the Czech Republic (crude 394 deaths per 100,000 inhabitants). In the United Kingdom, 182,266 COVID-19-related deaths were reported between 29 March 2020 and 31 December 2022 (crude 270 deaths per 100,000 inhabitants). Age-standardised rates and crude rates are similar, as demonstrated by Pizzato et al. [7]. The lower rate of COVID-19-related deaths in the United Kingdom could be explained by higher exposure of the Czech population to SARS-CoV-2 virus in early and late 2021 as described in the Czech national seroprevalence study [16, 17], although the potentially different mode of ascertaining the cause of death should also be considered.

In line with previous studies, our results showed that the risk of death due to COVID-19 was 30% higher in men than in women [4, 18]. The all-cause mortality data also showed significant proportion of excess deaths in the male population. The difference between male and female life expectancy in the Czech Republic was 5.8 years (76.3 for men and 82.1 for women) in 2019 and 6.4 years (74.1 for men and 80.5 for women) in 2021. On the other hand, the gap narrowed to 5.9 years in 2022, very similar to pre-pandemic level.

An important finding is that the COVID-19 pandemic did not affect mortality in children and adolescents. There are several potential explanations. Transcriptomic studies have shown that children have lower levels and activity of membrane-bound angiotensin converting enzyme 2 (ACE2) than adults. Membrane-bound ACE2 is required for SARS-CoV-2 to enter mucosal cells and cause COVID-19 [19]. On the other hand, children have higher plasmatic level of circulating soluble ACE2, which tends to bind to the spike protein of SARS-CoV-2. This could play a role in reducing the possibility of the virus to bind to the membrane bound ACE2 receptor and prevent severe systemic complications [20].

Excess pandemic mortality was found in adults and the elderly compared to pre-pandemic years. In the Czech Republic, excess mortality in the 50–64 age group only increased in 2021, rising by 16%. In the UK, the rate rose in this age group across all three years of the pandemic: by 19% in both 2020 and 2021, and by 12% in 2022. This may reflect differences in healthcare for this economically active population. Among those aged 75–84, the UK experienced high excess mortality only in 2020 (21%), which decreased thereafter [15, 21]. By contrast, the Czech Republic had significantly higher excess mortality in this age group throughout the pandemic: 24% in 2020, 39% in 2021 and 19% in 2022.We estimated the excess deaths due to specific cause of deaths in pandemic period compared to the average number of deaths in the 5 pre-pandemic years. No considerable excess deaths were found for the groups of diseases that have long been reported as the main categories of causes of death (circulatory diseases, cancers and respiratory diseases). These results could be interpreted as suggesting that the Czech healthcare system was prepared to adequately control the consequences of the pandemic as well as to provide ordinary healthcare.

On the other hand, several selected causes of deaths exhibited marked changes against the pre-pandemic years. Significant reductions in mortality were seen for ischaemic heart disease and cerebrovascular disease in all three years of the pandemic. This may be related to recent marked improvements in the standard treatment of ischaemic stroke using intravenous thrombolysis (IVT) and, more recently, endovascular therapy (EVT). Since 2016, the Czech Republic has led Europe in the number of EVT procedures performed per 1,000 ischaemic strokes. EVT is one of the most effective treatments in modern medicine [22, 23]. As a result, stroke mortality is decreasing in the Czech Republic.

On the other hand, several selected causes of death (heart failure, diabetes mellitus and Parkinson’s disease) increased by more than 20% when compared with pre-pandemic years. It has been suggested that these conditions are related to mitochondrial dysfunction [14]. The human heart has the highest content of mitochondria of any tissue (25–30% of cell volume) [24]. Progression to heart failure is associated with a decrease in energy reserve capacity, which finally reaches a threshold. Once this threshold is exceeded, compensatory mechanisms can no longer support the decreasing energy supply [25]. The crucial role of mitochondria in diabetes is evident from the fact that in β cells, around 80% of glucose oxidation takes place in mitochondria. Oxidative stress and increased inflammation are associated with blood glucose levels and glucose intolerance and accelerate insulin resistance [26, 27]. In addition, people with insulin resistance are at increased risk of heart failure [28]. Mitochondrial dysfunction and damage also contribute to several pathological cascades implicated in Parkinson’s disease [29]. Many affected at-risk neurons show elevated levels of ROS (reactive oxygen species) and mitochondrial oxidative stress [29, 30]. Compared with the last five years before the pandemic, pandemic mortality due to Parkinson’s disease was 38% higher in 2020, 49% higher in 2021 and 46% higher in 2022 in the Czech Republic.

Excess deaths from neurodegenerative diseases were previously reported in the US, where the observed/expected ratio for Alzheimer’s disease and related dementias was 1.26 in the first year of the pandemic; by 2021, this ratio fell to 1.05 [31]. Higher mortality from diabetes was observed in many countries [5, 8, 21], along with an increased diabetes incidence during the COVID-19 pandemic [32]. Reports regarding heart failure are inconclusive; while Pearson-Stuttard reported 20% excess heart failure deaths in the UK [21], a US study showed excess deaths from all cardiovascular diseases except heart failure [33]. This may be due to several factors, including, at the biomolecular level, a synergistic dysfunction of the endothelium and the mitochondria as well inaccurate rules for the counting of deaths from heart failure.

Home deaths were persistently elevated throughout the COVID-19 pandemic, while hospital deaths declined to pre-pandemic levels by 2022. The reasons for the persistently elevated home deaths during the pandemic are unclear. We can hypothesise that hospitals were not seen as a safe place because of the fear of COVID-19 infection. As our data showed, the capacity issue was only relevant in 2020 and 2021. Our findings are consistent with data from the United Kingdom, which showed a similar pattern with an increased proportion of pandemic deaths occurring at home [15, 34].

### Strengths and limitations

There are several limitations of this study. First, our study did not use statistical models that include multiple factors known to affect mortality, such as temporal trends, seasonality, heat waves, and influenza outbreaks, because such data were not available. To estimate the expected number of deaths, we use mortality data for a period of 5 years before the COVID-19 pandemic. We believe that a 5-year baseline period allows us to comprehensively assess mortality trends and to mitigate/eliminate the effects of perturbations such as heat waves and influenza outbreaks. Second, our results could be imprecise due to the migration from Ukraine. In 2022, more than 330,000 Ukrainians fled the war to the Czech Republic, 80% of them women under 50, children and teenagers. This may have affected the number and classification of deaths in national statistics in 2022. Third, because of the ecological study design, our study is limited in its ability to demonstrate the causal effect of COVID-19. Finally, proper causes of death certification during the COVID-19 pandemic became even more difficult for physicians than in pre-pandemic period. There could be many reasons for this, such as physician error or omission (due to overwork), the inability to test every patient for COVID-19, and finally the time lag between infection, recovery, and cause of death.

Despite these drawbacks, a notable strength of our study is that we performed these analyses using officially reported nationwide mortality data for all three COVID-19 pandemic years. This means that our results cover pandemic periods when the health system was overwhelmed, the vaccination period, as well as the post-vaccination period when different SARS-CoV-2 variants circulated from those observed in the first pandemic year. Another major strength of our study is the stratification of our results according to specific causes of death as well as according to individual characteristics.

## Conclusions

The full impact of the COVID-19 pandemic on mortality in the Czech Republic was greater than indicated by the reported deaths due to COVID-19, with about a quarter of the total excess being due to causes other than COVID-19. The excess mortality was evident in the productive age groups, and it was more pronounced in men than in women. Mortality from some major causes of death (cardiovascular diseases, cancer, and respiratory diseases other than COVID-19) declined during the pandemic, while some causes of death (heart failure, diabetes mellitus and neurodegenerative diseases) showed a marked increase, potentially suggesting a role of mitochondrial dysfunction as a possible mechanism. 

## Supplementary Information

Supplementary Material 1.

## Data Availability

Data for this study has been provided by Institute of Health Information and Statistics of the Czech Republic (https://www.uzis.cz/index-en.php). Due to national legal regulations (Sect.  73 of Czech Act No. 372/2011 Coll., on Health Services) and European data protection laws (GDPR), the authors are strictly prohibited from redistributing or publicly hosting individual-level health records. However, the data are available to independent researchers for replication purposes upon reasonable request. Requests for data access must be submitted directly to the IHIS CR via their official data application portal (available at: uzis.cz) or by email at uzis@uzis.cz, subject to the standard institutional approval protocol.
