# Supplementary material for: Excess mortality related to the COVID-19 pandemic by age, sex, and cause of death in the Czech Republic
Source: BMC Public Health. 2026 Sep 10;26:3145. doi: 10.1186/s12889-026-29300-1 (PMC13721334; doi:10.1186/s12889-026-29300-1)
Supplement: Supplementary file 1 [file 12889_2026_29300_MOESM1_ESM.docx]

**Supplementary table 1 Reported deaths in different age groups during the pandemic period in the Czech Republic and relative risk of death in different age groups (95% confidence interval) compared to the last five pre-pandemic years 2015-2019**

| Total reported deaths | | | | | | | |
| --- | --- | --- | --- | --- | --- | --- | --- |
| **Age group** | **Avg. annual rate 2015-2019** | **Reported deaths 2020** | **RR 2020 vs. prepandemic** | **Reported deaths 2021** | **RR 2021 vs. prepandemic** | **Reported deaths 2022** | **RR 2022 vs. prepandemic** |
| <12 m | 295 | 249 | 0.84 (0.71-0.99) | 246 | 0.84 (0.71-1.00) | 230 | 0.77 (0.64-0.91) |
| 1-9 y | 117 | 100 | 0.85 (0.65-1.11) | 100 | 0.86 (0.66-1.13) | 112 | 0.94 (0.72-1.22) |
| 10-24 y | 447 | 367 | 0.82 (0.71-0.94) | 391 | 0.88 (0.77-1.01) | 472 | 1.04 (0.91-1.18) |
| 25-49 y | 4,527 | 4,841 | 1.06 (1.02-1.11) | 5,456 | 1.22 (1.17-1.23) | 4,851 | 1.05 (1.01-1.09) |
| 50-64 y | 14,606 | 14,488 | 0.98 (0.96-1.01) | 16,706 | 1.16 (1.13-1.18) | 13,299 | 0.89 (0.87-0.91) |
| 65-74 y | 26,258 | 30,045 | 1.14 (1.12-1.15) | 34,612 | 1.33 (1.31-1.35) | 27,073 | 1.01 (0.99-1.03) |
| 75-84 y | 31,553 | 39,348 | 1.24 (1.22-1.26) | 43,444 | 1.39 (1.37-1.41) | 38,399 | 1.19 (1.18-1.21) |
| >=85 y | 33,327 | 39,851 | 1.19 (1.17-1.20) | 38,936 | 1.18 (1.16-1.20) | 35,783 | 1.05 (1.04-1.07) |
| **Total** | **111,130** | **129,289** | **1.15 (1.15-1.16)** | **139,891** | **1.27 (1.26-1.28)** | **120,219** | **1.06 (1.05-1.07)** |
| Reported deaths without deaths from COVID-19 | | | | | | | |
| **Age group** | **Avg. annual rate 2015-2019*** | **Reported deaths 2020** | **RR 2020 vs. prepandemic** | **Reported deaths 2021** | **RR 2021 vs. prepandemic** | **Reported deaths 2022** | **RR 2022 vs. prepandemic** |
| <12 m | 295 | 249 | 0.84 (0.71-0.99) | 242 | 0.83 (0.70-0.98) | 230 | 0.77 (0.64-0.91) |
| 1-9 y | 117 | 100 | 0.85 (0.65-1.11) | 96 | 0.83 (0.63-1.09) | 110 | 0.92 (0.71-1.20) |
| 10-24 y | 447 | 365 | 0.81 (0.71-0.93) | 385 | 0.87 (0.76-1.00) | 467 | 1.02 (0.90-1.17) |
| 25-49 y | 4,527 | 4,703 | 1.03 (0.99-1.07) | 4,853 | 1.08 (1.04-1.13) | 4,752 | 1.03 (0.99-1.07) |
| 50-64 y | 14,606 | 13,765 | 0.94 (0.91-0.96) | 13,766 | 0.95 (0.93-0.97) | 12,854 | 0.86 (0.84-0.88) |
| 65-74 y | 26,258 | 27,668 | 1.05 (1.03-1.06) | 27,290 | 1.05 (1.03-1.07) | 25,815 | 0.96 (0.95-0.98) |
| 75-84 y | 31,553 | 35,330 | 1.11 (1.09-1.13) | 34,547 | 1.11 (1.09-1.12) | 36,221 | 1.13 (1.11-1.14) |
| >=85 y | 33,327 | 36,570 | 1.09 (1.07-1.11) | 33,257 | 1.01 (0.99-1.02) | 33,735 | 0.99 (0.98-1.01) |
| **Total** | **111,130** | **118,750** | **1.06 (1.05-1.07)** | **114,436** | **1.04 (1.03-1.05)** | **114,184** | **1.01 (1.00-1.02)** |

**Supplementary table 2 Mortality rates (per 100,000) and relative risk of death from specific causes of death**

| **ICD-10** | **Cause of death** | **Average. annual rate  2015- 2019** | **2020**  **rate** | **RR 2020 vs. prepandemic** | **2021**  **rate** | **RR 2021 vs. prepandemic** | **2022**  **rate** | **RR 2022 vs. prepandemic** |
| --- | --- | --- | --- | --- | --- | --- | --- | --- |
| I00-I99 | All diseases of the circulatory system | 460 | 479 | 1.04 (1.03-1.06) | 455 | 0.99 (0.98-1.00) | 427 | 0.93 (0.92-0.94) |
| I20-I25 | Ischaemic heart diseases | 227 | 218 | 0.96 (0.95-0.98) | 208 | 0.92 (0.90-0.93) | 183 | 0.81 (0.79-0.82) |
| I60-I69 | Cerebrovascular diseases | 80 | 71 | 0.89 (0.86-0.92) | 68 | 0.85 (0.82-0.88) | 64 | 0.80 (0.77-0.82) |
| I50 | Heart failure | 46 | 63 | 1.36 (1.31-1.42) | 62 | 1.35 (1.30-1.40) | 67 | 1.46 (1.41-1.51) |
| C00-C97 | Malignant neoplasms | 259 | 262 | 1.01 (1.00-1.03) | 257 | 0.99 (0.98-1.01) | 254 | 0.98 (0.97-0.99) |
| J00-J99 | Diseases of the respiratory system | 74 | 77 | 1.05 (1.02-1.08) | 74 | 1.00 (0.97-1.03) | 78 | 1.06 (1.03-1.10) |
| J12-J18 | Pneumonia | 29 | 35 | 1.21 (1.15-1.27) | 34 | 1.15 (1.10-1.21) | 35 | 1.19 (1.13-1.25) |
| J40-J47 | Chronic lower respiratory diseases | 33 | 32 | 0.96 (0.90-0.99) | 31 | 0.93 (0.89-0.97) | 33 | 0.99 (0.94-1.04) |
| K00-K93 | Diseases of the digestive system | 45 | 48 | 1.06 (1.02-1.10) | 51 | 1.13 (1.09-1.18) | 50 | 1.11 (1.07-1.16) |
| K70-K77 | Diseases of liver | 22 | 23 | 1.08 (1.02-1.14) | 26 | 1.20 (1.14-1.27) | 25 | 1.17 (1.11-1.24) |
| E10-E15 | Diabetes mellitus | 37 | 47 | 1.25 (1.20-1.31) | 48 | 1.30 (1.25-1.36) | 45 | 1.20 (1.15-1.25) |
| N00-N39 | Diseases of the genitourinary system | 15 | 17 | 1.18 (1.10-1.26) | 19 | 1.32 (1.23-1.41) | 18 | 1.20 (1.12-1.28) |
| G20 | Parkinson disease | 4 | 5 | 1.38 (1.22-1.57) | 6 | 1.49 (1.31-1.69) | 5 | 1.46 (1.29-1.66) |
| G30-G32 | Other neurodegenerative diseases | 18 | 25 | 1.35 (1.27-1.43) | 22 | 1.21 (1.14-1.28) | 24 | 1.32 (1.24-1.40) |
| X60-X84 | Intentional self-harm | 13 | 11 | 0.91 (0.85-0.99) | 12 | 0.93 (0.86-1.00) | 12 | 0.96 (0.89-1.04) |

**Supplementary table 3 Mortality rates (per 100,000) and relative risk of death from specific causes of death by sex**

| **Cause of death** | **Average. annual rate  2015- 2019** | **2020**  **rate** | **RR 2020 vs. prepandemic** | **2021**  **rate** | **RR 2021 vs. prepandemic** | **2022**  **rate** | **RR 2022 vs. prepandemic** |
| --- | --- | --- | --- | --- | --- | --- | --- |
| All diseases of the circulatory system – male | 436 | 460 | 1.06 (1.04-1.08) | 443 | 1.02 (1.00-1.04) | 410 | 0.94 (0.92-0.96) |
| All diseases of the circulatory system – female | 483 | 498 | 1.03 (1.01-1.05) | 467 | 0.97 (0.95-0.98) | 444 | 0.92 (0.90-0.94) |
| Ischaemic heart diseases – male | 227 | 225 | 0.99 (0.96-1.01) | 218 | 0.96 (0.94-0.99) | 189 | 0.83 (0.81-0.86) |
| Ischaemic heart diseases – female | 227 | 212 | 0.94 (0.91-0.96) | 198 | 0.87 (0.85-0.90) | 177 | 0.78 (0.76-0.80) |
| Cerebrovascular diseases – male | 69 | 64 | 0.93 (0.88-0.97) | 60 | 0.87 (0.83-0.92) | 57 | 0.83 (0.79-0.87) |
| Cerebrovascular diseases – female | 90 | 77 | 0.86 (0.82-0.89) | 75 | 0.83 (0.80-0.87) | 70 | 0.77 (0.74-0.81) |
| Heart failure – male | 43 | 58 | 1.37 (1.29-1.44) | 58 | 1.35 (1.28-1.43) | 60 | 1.41 (1.34-1.49) |
| Heart failure – female | 49 | 67 | 1.36 (1.30-1.43) | 66 | 1.35 (1.28-1.42) | 74 | 1.50 (1.43-1.57) |
| Malignant neoplasms – male | 291 | 295 | 1.01 (0.99-1.04) | 285 | 0.98 (0.96-1.00) | 283 | 0.97 (0.95-0.99) |
| Malignant neoplasms – female | 227 | 230 | 1.01 (0.99-1.04) | 231 | 1.01 (0.99-1.04) | 226 | 1.00 (0.97-1.02) |
| Diseases of the respiratory system – male | 82 | 89 | 1.08 (1.04-1.13) | 84 | 1.02 (0.98-1.07) | 88 | 1.07 (1.03-1.12) |
| Diseases of the respiratory system – female | 66 | 67 | 1.01 (0.97-1.06) | 63 | 0.96 (0.92-1.01) | 69 | 1.06 (1.01-1.10) |
| Pneumonia – male | 31 | 39 | 1.29 (1.21-1.38) | 38 | 1.23 (1.15-1.31) | 38 | 1.26 (1.18-1.34) |
| Pneumonia – female | 28 | 32 | 1.12 (1.05-1.20) | 30 | 1.07 (1.00-1.15) | 31 | 1.12 (1.04-1.19) |
| Chronic lower respiratory diseases – male | 40 | 37 | 0.94 (0.89-1.00) | 36 | 0.92 (0.87-0.98) | 38 | 0.96 (0.90-1.02) |
| Chronic lower respiratory diseases – female | 27 | 26 | 0.95 (0.88-1.02) | 26 | 0.94 (0.87-1.01) | 28 | 1.04 (0.96-1.11) |
| Diseases of the digestive system – male | 52 | 56 | 1.08 (1.02-1.14) | 60 | 1.16 (1.10-1.22) | 59 | 1.13 (1.07-1.19) |
| Diseases of the digestive system – female | 38 | 39 | 1.03 (0.97-1.09) | 42 | 1.10 (1.03-1.17) | 42 | 1.09 (1.03-1.16) |
| Diseases of liver – male | 29 | 32 | 1.08 (1.01-1.16) | 35 | 1.19 (1.11-1.28) | 35 | 1.19 (1.10-1.27) |
| Diseases of liver – female | 14 | 15 | 1.07 (0.97-1.18) | 17 | 1.22 (1.11-1.34) | 16 | 1.15 (1.04-1.26) |
| Diabetes mellitus – male | 35 | 44 | 1.27 (1.19-1.35) | 49 | 1.40 (1.32-1.49) | 43 | 1.24 (1.17-1.32) |
| Diabetes mellitus – female | 40 | 49 | 1.24 (1.17-1.31) | 48 | 1.21 (1.15-1.29) | 46 | 1.17 (1.10-1.23) |
| Diseases of the genitourinary system – male | 13 | 17 | 1.27 (1.15-1.40) | 18 | 1.36 (1.23-1.50) | 17 | 1.28 (1.16-1.41) |
| Diseases of the genitourinary system – female | 16 | 18 | 1.10 (1.00-1.21) | 21 | 1.28 (1.17-1.40) | 18 | 1.13 (1.03-1.23) |
| Parkinson disease – male | 5 | 6 | 1.30 (1.10-1.54) | 6 | 1.41 (1.20-1.67) | 7 | 1.46 (1.24-1.72) |
| Parkinson disease – female | 3 | 4 | 1.51 (1.23-1.85) | 5 | 1.60 (1.31-1.96) | 4 | 1.47 (1.10-1.80) |
| Other neurodegenerative diseases – male | 13 | 18 | 1.33 (1.20-1.46) | 15 | 1.11 (1.00-1.23) | 16 | 1.20 (1.09-1.33) |
| Other neurodegenerative diseases – female | 24 | 32 | 1.36 (1.26-1.46) | 30 | 1.26 (1.17-1.36) | 32 | 1.38 (1.28-1.48) |
| Intentional self-harm – male | 20 | 19 | 0.93 (0.86-1.02) | 19 | 0.91 (0.84-0.99) | 19 | 0.95 (0.87-1.03) |
| Intentional self-harm – female | 5 | 4 | 0.83 (0.69-0.99) | 5 | 1.00 (0.84-1.19) | 5 | 1.03 (0.87-1.22) |
